# A novel framework for human factors analysis and classification system for medical errors (HFACS-MES)—A Delphi study and causality analysis

**DOI:** 10.1371/journal.pone.0298606

**Published:** 2024-02-23

**Authors:** Mahdi Jalali, Ehsanollah Habibi, Nima Khakzad, Shapour Badiee Aval, Habibollah Dehghan

**Affiliations:** 1 Department of Occupational Health Engineering, Student Research Committee, School of Public Health, Isfahan University of Medical Sciences, Isfahan, Iran; 2 Department of Occupational Health Engineering, School of Public Health, Isfahan University of Medical Sciences, Isfahan, Iran; 3 School of Occupational and Public Health, Toronto Metropolitan University, Toronto, Canada; 4 Department of Complementary and Chinese Medicine, School of Persian and Complementary Medicine, Mashhad University of Medical Sciences, Mashhad, Iran; Sunway University, MALAYSIA

## Abstract

The healthcare system (HCS) is one of the most crucial and essential systems for humanity. Currently, supplying the patients’ safety and preventing the medical adverse events (MAEs) in HCS is a global issue. Human and organizational factors (HOFs) are the primary causes of MAEs. However, there are limited analytical methods to investigate the role of these factors in medical errors (MEs). The aim of present study was to introduce a new and applicable framework for the causation of MAEs based on the original HFACS. In this descriptive-analytical study, HOFs related to MEs were initially extracted through a comprehensive literature review. Subsequently, a Delphi study was employed to develop a new human factors analysis and classification system for medical errors (HFACS-MEs) framework. To validate this framework in the causation and analysis of MEs, 180 MAEs were analyzed by using HFACS-MEs. The results showed that the new HFACS-MEs model comprised 5 causal levels and 25 causal categories. The most significant changes in HFACS-MEs compared to the original HFACS included adding a fifth causal level, named "extra-organizational issues", adding the causal categories "management of change" (MOC) and "patient safety culture" (PSC) to fourth causal level", adding "patient-related factors (PRF)" and "task elements" to second causal level and finally, appending "situational violations" to first causal level. Causality analyses among categories in the HFACS-MEs framework showed that the new added causal level (extra-organizational issues) have statistically significant relationships with causal factors of lower levels (Φc≤0.41, p-value≤0.038). Other new causal category including MOC, PSC, PRF and situational violations significantly influenced by the causal categories of higher levels and had an statistically significant effect on the lower-level causal categories (Φc>0.2, p-value<0.05). The framework developed in this study serves as a valuable tool in identifying the causes and causal pathways of MAEs, facilitating a comprehensive analysis of the human factors that significantly impact patient safety within HCS.

## Introduction

The HCS stands as one of society’s most critical and essential structures, encompassing various sectors across different countries. Among its key components, hospitals hold a pivotal position in safeguarding human health. Charged with the mission of delivering secure and effective medical services to patients, hospitals occasionally become sources of harm. Despite the integration of advanced technologies and care, MEs give rise to diverse complications and fatalities, inflicting significant costs upon both patients and society [1]. As per the definition, a medical error refers to an unintended action stemming from negligence, resulting in adverse outcomes during medical procedures. In essence, a medical error represents an action or decision that deviates from the standards established by the HCS [2,3].

Previous studies have reported that the approximately ten percent of patients have been affected by at least one adverse event [4]. Countries with less income report higher rates of adverse events [5]. Recent conservative estimates highlight patient injury as the fourteenth leading cause of death worldwide. According to the World Health Organization (WHO), approximately 421 million hospitalizations are annually carried out globally, with approximately 42.7 million instances of adverse events occurring during these hospital stays [6]. Studies reveal that, on average, 1 in 10 patients experiences an adverse event during hospital care in high-income countries [7]. Estimates from low- and middle-income countries suggest that one in four patients suffer harm, leading to 134 million adverse events approximately 2.6 million deaths annually due to unsafe hospital care. Globally, the financial toll of medication errors is projected at $42 billion per year, accounting for roughly 1% of global healthcare spending [6]. Recent projections indicate that the societal cost of patient harm is in the range of 1 to 2 trillion dollars annually. Following the human capital approach, eliminating harm could potentially boost global economic growth by over 0.7% annually [8]. Consequently, ensuring patient safety and preventing MEs and MAEs in hospital settings have emerged as critical global priorities.

The HCS remains susceptible to errors owing to its intricate and high-risk nature [9,10]. Its complexity stems from various factors. Firstly, healthcare constitutes a multifaceted domain necessitating intricate procedures and sophisticated equipment [11]. Secondly, it relies on interconnected and interdependent components, often requiring diverse personnel from different departments to collaborate in delivering specific treatments [12]. Errors originating in one component can readily impact others, with repercussions often proving unpredictable, especially if the affected component is distant from the initial error’s origin. Thirdly, the system’s components are tightly integrated, leading to rapid error propagation from one component to another [13]. Fourth, the HCS was designed and is operated by human beings, and human beings cannot predict all the possible effects of decisions or actions within the system [14]. Consequently, HCS to achieve a state of high reliability, it should adopt an advanced approach to detecting and comprehending MEs and MAEs [15]. However, regrettably, the sector has yet to attain the status of high reliability witnessed in other sectors (such us aviation industry, nuclear industry, process industry and etc) [16].

**Fig 1:**
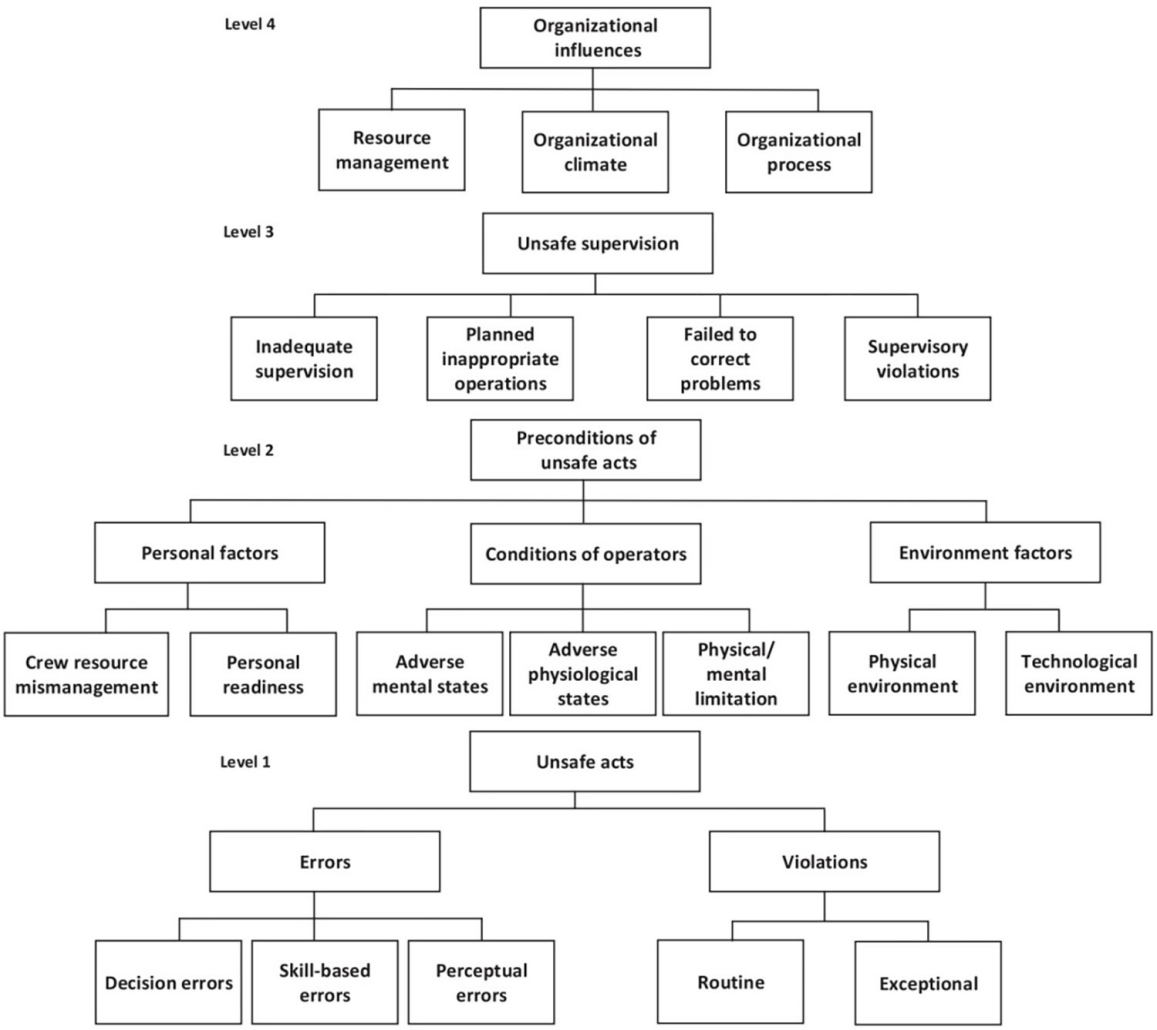
The framework of the original human factor analysis and classification system [22].

One of the approaches borrowed from high reliability organizations for uncovering and comprehending MEs and adverse events in the HCS is the root cause analysis (RCA). This structured and retrospective method is designed to identify the actual causes of a problem and devise strategies to eliminate them by focusing on the root causes [17]. Despite its widespread application and recent advancement, the use of this method has not resulted in a significant progress in ensuring patient safety [18]. HFACS framework has been proposed as an effective tool to enhance patient safety and mitigate MEs in the HCS. Initially introduced by Chapel and Wigman (2001) to address the limitations of the Swiss Cheese method, the HFACS framework features a hierarchical structure comprising 19 causal categories organized into four distinct levels. These levels encompass unsafe acts, preconditions for unsafe acts, unsafe supervision, and organizational influences (Fig 1). Through the examination of numerous incident reports, HFACS was meticulously tailored to identify underlying internal deficiencies and bridge the gap between theory and practice within the Swiss Cheese model. The HFACS framework proves effective at scrutinizing human factors, particularly with regard to safety culture, management commitment, safety leadership, organizational shortcomings, technical deficiencies in outdated equipment, and gaps in knowledge or operator competency [19]. However, its current design does not allow for the simultaneous diagnosis of extra-organizational shortcomings and certain specific issues within the HCS, including incidents stemming from patient and caregiver behaviour [19–21].

In recent years, several studies have proposed modifications to the HFACS with the aim of adapting it for the HCS. However, none of these studies have presented the revised structure using a transparent scientific and systematic methodology. Despite the alterations made to the original HFACS framework, the causal pathways, the interrelation between the newly introduced levels, and the validity of the proposed framework still remain ambiguous [15,21,23,24]. Therefore, this study endeavours to introduce an improved and practical framework for understanding the causality of MAEs based on the foundational principles of HFACS. The development of this new framework has been informed by an exhaustive literature review and expert insights, adhering to a structured and scientific approach. Its utility in uncovering the root causes of MAEs has been validated through the systematic determination of causal pathways. The adoption of this improved HFACS framework promises significant benefits, particularly in the analysis of MAEs within the HCS.

## Material and methods

**Fig 2:**
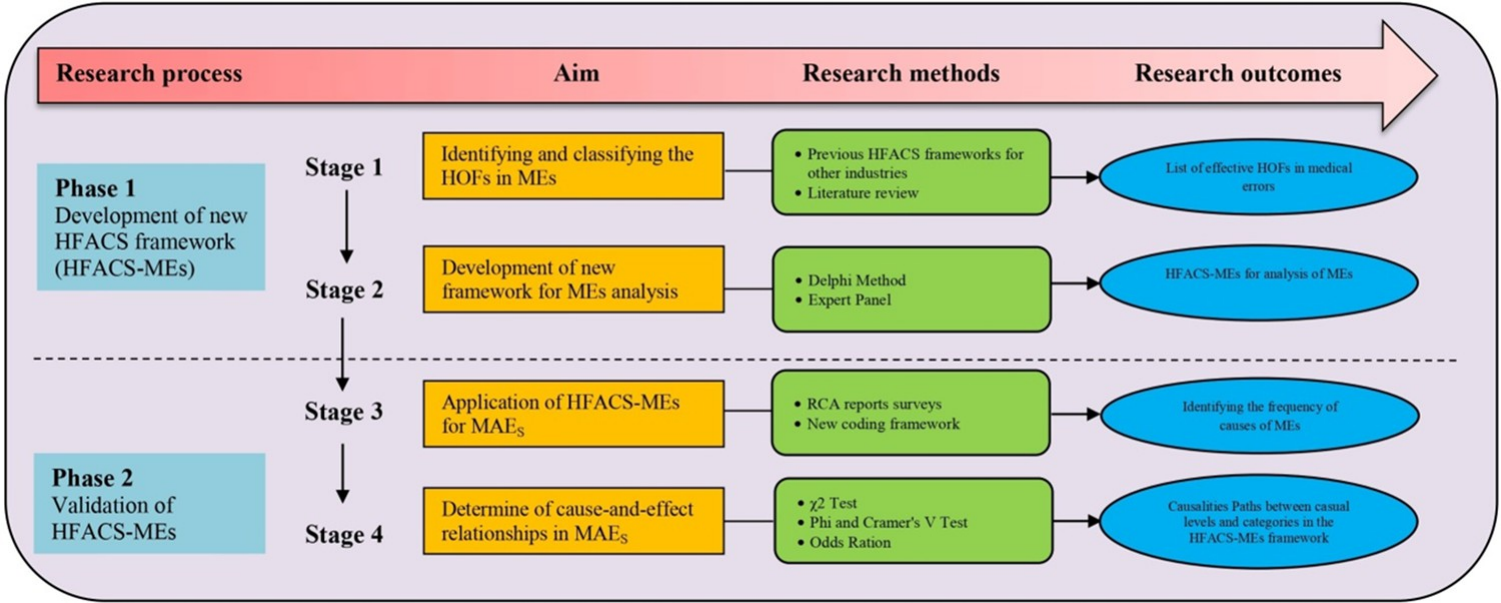
Study framework for development of HFACS-MEs.

This descriptive-analytical study was conducted in Iranian hospitals in 2023. Prior to commencing the investigation, ethical clearance was obtained from the ethical committee of Isfahan University of medical sciences (IR.MUI.RESEARCH.REC.1400.371). Before the study, written consent forms were prepared and provided to the participants. All participants completed and signed the informed consent forms. The study was conducted in two phases. Firstly, we developed an improved Human Factors Analysis and Classification System for Medical Errors (HFACS-MEs) from the original HFACS proposed by Shappell and Wiegmann [22]. Subsequently, validation of HFACS-MEs was done. In order to verification of this framework in the coding, causation and classification of MEs, 180 MAEs (analysed by RCA) were analysed and the cause-and-effect relationships of these events were determined using HSACS-MEs. The step-by-step development of the framework is illustrated in Fig 2, while further elaboration on these stages has been provided in the subsequent sections.

### Phase 1. Development of the new HFACS framework

During this phase, HFACS-MEs were formulated through a combination of expert insights and empirical evidence. Initially, we identified potential HOFs associated with MEs. Subsequently, HFACS-MEs was constructed employing the Delphi method. The development process of the new HFACS-MEs framework followed a four-step approach. Firstly, we compiled an initial inventory of potential HOFs through an exhaustive literature search. Secondly, we conducted an initial round of questionnaires, requesting the expert panel members to assess the appropriateness of each potential HOF based on a rating scale. Thirdly, a face-to-face expert panel meeting was convened, encouraging panel members to deliberate on the suitability of the identified HOFs based on the scores obtained in the initial questionnaire round. Finally, a second round of questionnaires was administered to consolidate the definitive set of HOFs for incorporation into the HFACS-MEs framework.

#### Stage 1. Identification of potential HOFs related to MAEs

At this juncture, HOFs associated with MAEs were systematically identified. To achieve this, a comprehensive examination of research focusing on the root causes of MEs and the shaping performance factors, which underlie human error, was conducted. Key references encompassed a range of human error evaluation methodologies, studies pertaining to the evaluation and causation of MEs, and the HOFs implicated in the occurrence of MEs. Moreover, insights from the development of HFACS in diverse industries were leveraged as crucial points of reference. The most important references used for the development of HFACS-MEs are presented in Table 1. Following the extraction of potential HOFs from the literature, a questionnaire encompassing these factors was formulated. Based on the framework of the original HFACS methodology, different factors were allocated to distinct categories within the questionnaire. In addition to the original HFACS method, new causal classes were included in the questionnaire. Upon the compilation of the questionnaire, the Delphi method was employed to ascertain the most significant and pertinent factors contributing to the occurrence of MAEs [25].

**Table 1:** The most important HFACS modifications in the development of HFACS-MEs.

#	Title of studies	Sector	Author(S)
**1**	A content analysis of contributing factors reported in serious incident investigation reports in hospital care	Healthcare	Peerally et al. [23]
**2**	Human factors approach for identifying latent failures in healthcare settings	Healthcare	Cohen et al. [1]
**3**	Systemic and human factors that contribute to medical error: A study of higher reliability	Healthcare	Burns et al [16]
**4**	The human factors analysis classification system (HFACS) applied to health care	Healthcare	Diller et al. [15]
**5**	Human factors analysis and classification system for the oil and gas industry (HFACS-OGI)	oil and gas industry	Theophilus et al. [19]
**6**	The development and validation of a human factors analysis and classification system for the construction industry	construction industry	Yong Chen [20]
**7**	Surgical never events and contributing human factors	Healthcare	Thiels et al. [21]
**8**	Investigations by acute-hospital staff: AcciMaps or HFACS?	Healthcare	Woodier et al. [26]
**9**	Human and organizational factors within the public sectors for the prevention and control of epidemic	Healthcare	Fu et al. [27]
**10**	Applying the human factors analysis and classification system to critical incident reports in anaesthesiology	Healthcare	Neuhaus et al. [28]
**11**	Human factor analysis and classification system for the oil, gas, and process industry	oil and gas industry	Yang J et al. [29]

The Delphi method is a process mostly used in research and economics, which aims to collect opinions on a particular research question or specific topic, to gain consensus. The opinions are collected from a group of experts that are not physically assembled, normally through questionnaires [30]. This technique was firstly developed in the 1950s for military actions as a systematic method to anticipate events. After it became a popular tool for business forecasting, and in the 2000s gained acceptance in the scientific community. At this method, the group facilitator selects a group of experts based on the topic being examined and sends them a questionnaire with instructions to comment on each topic based on their personal opinion, experience, or previous research. The facilitator groups the comments from the returned questionnaires and sends copies to each participant, along with the opportunity to comment further. At the end of this session, the questionnaires are returned to the facilitator, who decides if another round is necessary or if the results are ready for publishing. This process can be repeated multiple times until a general sense of consensus is reached [31]. This technique has recently been widely used in health sciences [32,33].

#### Stage 2. Development of HFACS-MEs

*2*.*1 First round of questionnaire survey*. A panel of experts was chosen to oversee the Delphi study. Selection criteria for these experts included a strong background in HFACS and RCA methodologies, substantial experience and expertise in the domain of accident and human error analysis, as well as active involvement in RCA committees and medical error analysis And the lack of familiarity of experts with each other. Our expert panel comprised fifteen individuals from diverse disciplines, including five occupational health and safety specialists, two primary care physicians, two medical specialists, two hospital quality improvement experts, three patient safety specialists, and one social medicine expert. We emailed the questionnaires and asked them to rate each potential HOFs based on the importance of causal levels and causal categories specified in the questionnaire to provide a model for classifying HOFs related to MEs based on a nine-point Likert Scale, with one indicating ‘‘definitely not appropriate” and nine indicating ‘‘definitely appropriate”. Scores equal or greater than 7 indicated high appropriateness. The percentage of agreement for each HOF was defined based on the proportion of experts who rated an HOF ≥ 7. Potential HOFs with a median score < 7 and those with a percentage of agreement ≤ 70% were allocated for discussion and modification during the subsequent expert panel meeting [34]. All questionnaires were coded and all answers were confidential, so that none of the experts knew each other’s opinions.

*2*.*2 Expert panel meeting*. All members of the panel attended an in-person meeting during which the outcomes of the first round of questionnaires were presented to facilitate a comprehensive discussion of the evaluated HOFs, with the intention of achieving a consensus. During this meeting, experts provided qualitative assessments and feedback on the identified HOFs. Additionally, panel members were encouraged to introduce new potential indicators, as deemed necessary. These fresh potential HOFs were qualitatively evaluated within the panel meeting and were subsequently subjected to quantitative assessment during the second round of questionnaires. This phase primarily focused on collecting insights pertaining to the potential renaming, relocation, removal, and addition of HOFs within the causal levels and categories.

*2*.*3 Second round of questionnaire survey and ranking*. Following the expert panel meeting, the list of all accepted, modified, and newly added potential HOFs was converted into the second questionnaire and sent to panel members again by e-mail for final appraisal. In this second round, respondents were asked to rate the potential HOFs in the same way as the first round. We selected HOFs with a median score ≥ 7 and percent agreement ≥ 70% as the final set of HFACS-MEs. Based on these results, the causal levels and causal categories of the new HFACS-MEs framework were developed based on the Delphi technique.

### Phase 2. Validation of the HFACS-MEs framework

In this phase, the HFACS-MEs were validated by analysing 180 MAEs. Next, cause-effect relationships between the different causal levels and categories of the HFACS-MEs framework were investigated quantitatively using valid statistical methods.

#### Stage 3. Coding Process using the new HFACS-MEs framework

Three occupational health and safety experts undertook the coding process based on the newly presented framework. Prior to this, the analysts underwent joint training in the utilization of the original HFACS method and possessed necessary expertise in employing this framework for causal analysis of incidents. Initially, two experts independently coded 180 RCA reports utilizing the specific nanocodes outlined in the new HFACS-MEs framework. Subsequently, the coding results for each RCA report were cross-validated between the two analysts. In cases where disparities emerged, discussions were revisited and continued until a consensus was reached. The agreed-upon results were then submitted to the third expert, who boasted extensive experience in root cause analysis and a track record of employing the original HFACS in coding MAEs. This specialist meticulously reviewed the findings of the preceding two analysts and oversaw the final stages of the coding process. Throughout the coding process, any discrepancies or uncertainties within the RCA reports were addressed through two primary approaches: First, by seeking assistance from the RCA committee members of the hospitals, and second, by conducting site visits and engaging with the hospital staff directly.

#### Stage 4. Causality analysis

In the original HFACS model, it is postulated that the factors at the upper levels, serving as latent influences on events, directly impact the lower tier and indirectly affect other lower tiers. Consequently, when delving into the causal factors of MAEs, it becomes feasible to scrutinize the influence of the causal categories of the upper tiers on the causal categories of the lower tier, thereby perpetuating this process from the highest tier to the lowest tier [20]. Hence, the causality among the levels of the HFACS-MEs, rooted in the original HFACS, follows a similar trajectory. Leveraging this framework, significant causal relationships within the categories can be unveiled through the computation of their correlations. To this end, the coded data underwent cross-tabulation, followed by the implementation of the chi-square test (χ^2^) to assess the statistical association between the causal categories of the higher and lower tiers.

However, as the χ^2^ test is a simple test of association these analyses were supplemented with further analyses using Phi Coefficient and Cramer’s V Correlation test (Φc) to determine the intensity of the relationship between significant factors. Phi is a measure of strength for an association between two categorical variables in a 2 × 2 contingency table. It varies between 0 and 1 without any negative values. The closer this value is to 1, the stronger the relationship and vice versa. Similar to Pearson’s r, a value close to 0 in phi coefficient means no association. However, a value larger than 0.25 implies a very strong relationship [35]. As a complementary method, using logistic regression, the odds ratio (OR) was also calculated. The odds ratio (OR) is commonly used to estimate how likely an event will occur compared to another event. In this study the OR test is used to estimate whether the failure of the factors at an upper level of HFACS- MEs would increase the failure of factors at a lower level, which is the case if the OR is greater than 1. [36]. First, statistical tests were performed for all pairs of groups of human factors, and statistically significant relationships with ORs > 1 were subsequently kept for further analysis and drawing of the causality path diagram. Data analysis was done using SPSS V-25 software. The significance level for all tests was set at α = 0.05.

## Results

After conducting an extensive literature review, a questionnaire comprising 5 causal levels and 36 causal categories was formulated and distributed to the experts. The designated levels encompassed level 1: unsafe acts (encompassing 6 causal categories); level 2: preconditions for unsafe acts (comprising 15 causal categories); level 3: supervision factors (involving 4 causal categories); level 4: organizational factors (consisting of 7 causal categories), and level 5: external factors (encompassing 4 causal categories). The results of the first and second rounds of Delphi are presented in Table 2.

**Table 2:** Assigned scores during the first and second questionnaire rounds.

Level	Causal categories		Round 1		Meeting decision	Round 2		Final decision
			Mean	Agreement %		Mean	Agreement %	
Outside factors	1	External environmental influences- Legislation & regulation	5.2	66	Deleted	×	×	×
	2	External environmental influences- Others	5.5	73	Deleted	×	×	×
	3	Extra-organisational issues- National Deficiencies	8.8	100	Accepted	9	100	Selected
	4	Extra-organisational issues- Others	8.2	100	Accepted	5.9	73	Deleted
	5	Extra-organisational issues- Legislation & regulation	NP	NP	Added	9	100	Selected
Organizational factors	1	Poor organisational process	8.5	100	Accepted	9	100	Selected
	2	Organizational process	8.4	93	Accepted	5.6	66	Deleted
	3	Organizational climate	5.8	73	Deleted	×	×	×
	4	Organizational culture	8.2	93	Accepted	2	26	Deleted
	5	Resource management	9	100	Accepted	9	100	Selected
	6	Management of change	9	100	Accepted	9	100	Selected
	7	Patient Safety Culture	8.6	100	Accepted	9	100	Selected
Unsafe supervision	1	Inadequate supervision (oversight)	9	100	Accepted	9	100	Selected
	2	Inadequate planning	9	100	Accepted	9	100	Selected
	3	Supervisory violation	9	100	Accepted	9	100	Selected
	4	Failure to address a known problem	9	100	Accepted	9	100	Selected
Preconditions for unsafe acts	1	Personal factors- Communication/ Coordination/planning	4.2	60	Deleted	×	×	×
	2	Personal factors- crew resource mismanagement	4	53	Deleted	×	×	×
	3	Team & Coordination- Communication	9	100	Modified	9	100	Selected
	4	Team & Coordination—Team dynamics	8.8	100	Modified	9	100	Selected
	5	Personal factors- personal readiness	7.6	86	Modified	9	100	Selected
	6	Personal factors- fitness for duty	4.5	66	Deleted	×	×	×
	7	Environmental factors—technological environment	5	73	Deleted	×	×	×
	8	Environmental factors—Person-machine interface	9	100	Accepted	9	100	Selected
	9	Environmental factors -physical environment	9	100	Accepted	9	100	Selected
	10	Environmental factors–Task elements	8.2	100	Accepted	7.8	80	Selected
	11	Environmental factors—Patient related factors	8.8	100	Accepted	9	100	Selected
	12	Environmental factors -Situational factors (lighting, noise, heat, emergency, …	3.8	53	Deleted	×	×	×
	13	Conditions of the operator-adverse mental state	9	100	Modified	9	100	Selected
	14	Conditions of the operator- adverse physiological state	9	100	Modified	9	100	Selected
	15	Conditions of the operator- chronic performance limitation	9	100	Modified	9	100	Selected
Unsafe acts	1	Errors-Decision Errors	9	100	Accepted	9	100	Selected
	2	Errors-Skill-based Errors	9	100	Accepted	9	100	Selected
	3	Errors-Perpetual Errors	9	100	Accepted	9	100	Selected
	4	Violations-Routine	9	100	Accepted	9	100	Selected
	5	Violations- Exceptional	9	100	Accepted	9	100	Selected
	6	Violations-situational	NP	NP	Added	7.6	73	Selected
	7	Violations- Acts of Sabotage	3.1	46	Deleted	×	×	×

× = Eliminated in the first round (Mean score < 7, Agreement < 70%).

NP = It was not initially presented but was added by the experts in the second round.

### Causal level 5- outside factors

In the first round of Delphi, the experts suggested that the name of level 5 be changed to "Extra-organisational issues". They also suggested that the causal category of external environmental influences should be removed and the causal category of legislation & regulation, and national deficiencies should be placed under extra-organizational issues. Consequently, this level was redefined as extra-organizational issues, encompassing 2 causal categories: legislation & regulation and national deficiencies.

### Causal level 4- Organizational factors

In the first round, according to the experts, the causal category "organizational climate" was removed from this level. In the second round, due to the overlap between "organizational culture" and PSC, it was decided to remove "organizational culture" and use the PSC alone. In addition, with the agreement of the experts, the causal category of MOC was added to this level for the first time.

### Causal level 3- unsafe supervision

In the first and second rounds of Delphi, all causal categories of "unsafe supervision" level were confirmed without change.

### Causal level 2- Preconditions for unsafe acts

With the opinion of experts in the first round, the causal factor "conditions of the operator" was changed to "healthcare providers". According to the experts’ opinion, "personal factors-communication/coordination/planning" and "personal factors-crew resource mismanagement" were eliminated in the first round. The opinion of the experts was that "communication" and "team dynamics" were better alternatives for these 2 causal categories. It was also suggested to remove the "personal factors" title and use the "team & coordination" title instead. In addition, in the first round of the causal categories "personal factors-fitness for duty" was eliminated with the opinion of the experts. They believed that the "personal factors-personal readiness" should be removed from the category of "personal factors". In the second round, this item was placed under the category "healthcare providers". The causal category of "environmental factors" also had the most changes in the Delphi survey. The causal category "person-machine interface" was found to be more appropriate than the causal category "environmental factors- technological environment" and was replaced in the first round. The causal categories "environmental factors- task elements" and "environmental factors- patient related factors (PRF)" were also added to this level. Finally, the causal category of "situational factors" was removed from this level with the opinion of experts in the first round. The experts believed that this causal factor can be integrated into the "physical environment".

### Causal level 1- unsafe acts

In this level, the causal category of "violations- acts of sabotage" was eliminated in the first round with the opinion of the experts. They believed that this class could be considered in the "violations-exceptional" category. Furthermore, the "violations-situational" category was added to this level in the first round as per the experts’ input and was subsequently approved by all the experts in the second round."

**Fig 3:**
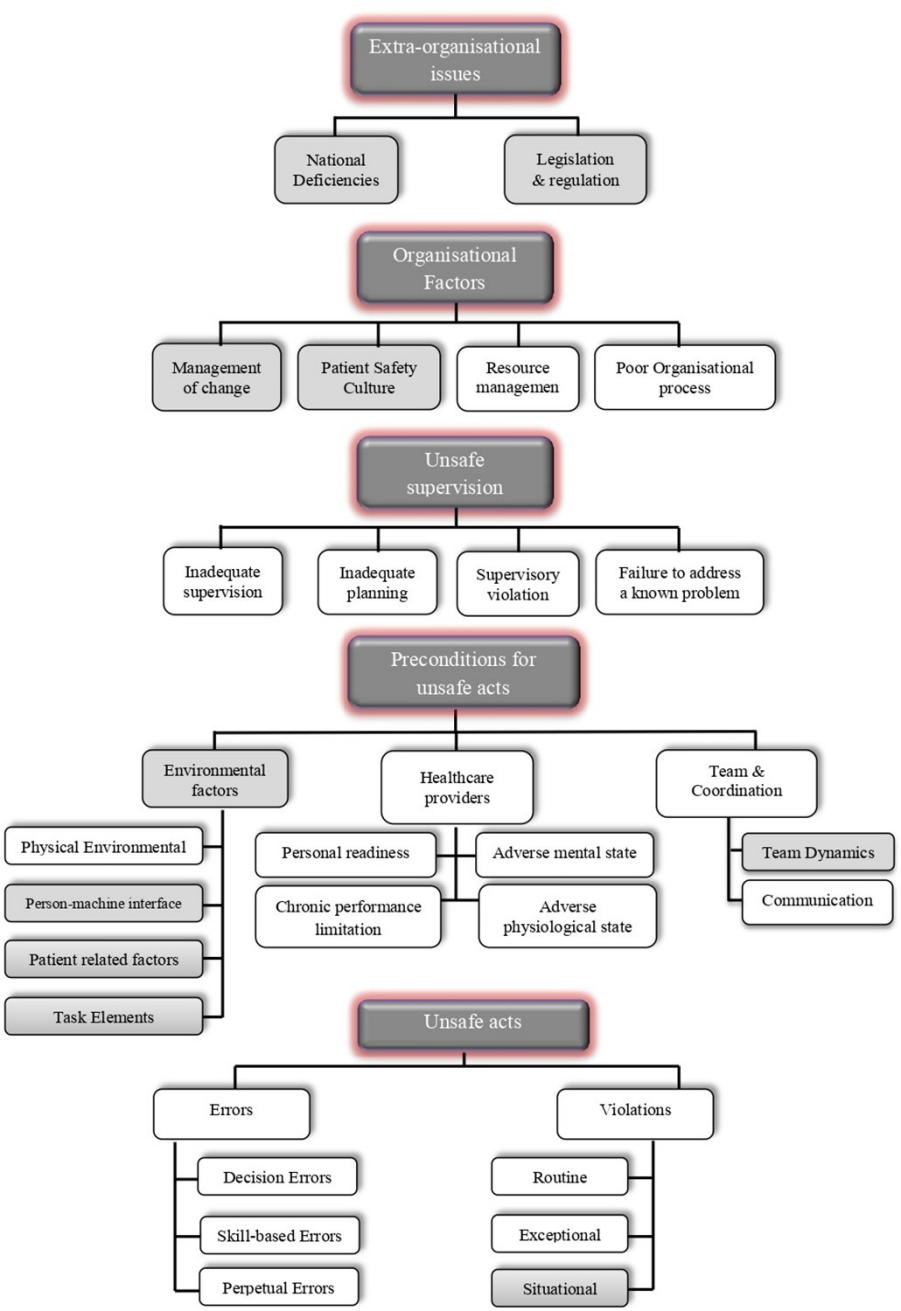
New framework of the proposed human factors analysis and classification system for the medical errors (HFACS-MEs).

Finally, the ultimate HFACS-MEs model comprised 5 causal levels and 25 causal categories. The definitive validated model resulting from the Delphi study is illustrated in Fig 3, where the added new causal level and new/modified causal categories are highlighted in grey boxes.

The relationship between the different levels of the HFACS-MEs framework and the correlation strength between pairs of human factors groups are shown in Table 3. It can be seen that 17 pairs of human factors in different levels are kept. These pairs have a significant causality from the perspective of the χ2 test.

**Table 3:** The pairs kept by the χ2 test (p-value < 0.05) and correlation strength between different HFACS-MEs levels.

#	Pairwise human factors	χ^2^ test	p-value	Φc
**Relationship between level 5 and Level 4 (5×4)**				
1	National Deficiencies × Management of change	11.660	0.001	0.360
2	Legislation & regulation × Patient Safety Culture	15.430	0.001	0.414
3	Legislation & regulation × Poor Organisational process	4.921	0.038	0.310
**Relationship between level 4 and Level 3 (4×3)**				
1	Management of change × Inadequate planning	6.970	0.008	0.278
2	Resource management × Supervisory violation	3.930	**0.048**	0.203
3	Poor Organisational process × Inadequate planning	3.880	**0.049**	0.206
4	Patient Safety Culture × Failure to address a known problem	4.371	0.041	0.301
**Relationship between level 3 and Level 2 (3×2)**				
1	Inadequate supervision × Communication	9.360	0.002	0.322
2	Inadequate planning × Patient related factors	9.405	0.002	0.323
**Relationship between level 2 and Level 1 (2×1)**				
1	Patient related factors × Decision Errors	4.896	0.027	0.233
2	Patient related factors × Situational violations	3.920	0.048	0.202
3	Person-machine interface × Skill-based Errors	6.881	0.009	0.277
4	Physical Environmental × Perpetual Errors	5.302c	0.017f	0.296
5	Chronic performance limitation × Situational violations	4.410	0.036	0.221
6	Adverse physiological state × Perpetual Errors	5.314c	0.018f	0.297
7	Adverse mental state × Perpetual Errors	3.892	0.049	0.201
8	Communication × Skill-based Errors	4.535	0.033	0.224

The relationship between the level of "extra-organizational issues" and the level of "organizational factors" showed that out of 8 possible relationships, 3 pairs of associations were significant between categories at adjacent levels. The results showed that there was a statistically significant relationship between "national deficiencies" and "MOC" (χ2 = 11.66, p-value = 0.001), "legislation & regulation" and "PSC" (χ2 = 15.43, p-value = 0.001) and "rules and regulations" with "organizational processes" (χ2 = 4.921, p-value = 0.038).

The relationship between the level of "organizational factors" and the level of "unsafe supervision" showed 4 pairs of associations were significant between the categories at these two adjacent levels. A statistically significant relationship was observed between "MOC" and "inadequate planning" (χ2 = 6.97, p value = 0.008), "PSC" and "failure to address a known problem" (χ2 = 4.371, p-value = 0.041), "resource management" and "supervisory violation" (χ2 = 3.93, p-value = 0.048) and "organizational processes" and "inadequate planning" (χ2 = 3.880, p-value = 0.049).

The relationship between the level of "unsafe supervision" and "preconditions for unsafe actions" showed that out of 40 possible relationships, 2 pairs of human factors have a significant relationship with each other. A statistically significant relationship was observed between "inadequate supervision" and "communication" (χ2 = 9.360, p-value = 0.002) and "inadequate planning" and "PRF" (χ2 = 9.405, p-value = 0.002).

The relationship between the level of "preconditions for unsafe acts" and the level of "unsafe acts" revealed that out of 60 possible relationships, 8 pairs of associations were significant between categories at adjacent levels. These were "PRF" versus "decision errors" (χ2 = 4.896, p-value = 0.027), "PRF" versus "situational violations" (χ2 = 3.920, p-value = 0.048), "person -machine interface" with "skill-based errors" (χ2 = 6.881, p-value = 0.009), "physical environment" versus "perceptual errors" (Continuity-corrected χ2 = 5.302, p-value = 0.017), "chronic performance limitation" with "situational violations" (χ2 = 4.410, p-value = 0.036), "adverse physiological state" versus "perceptual errors" (Continuity-corrected χ2 = 5.314, p-value = 0.018), "adverse mental state" with "Perceptual errors" (χ2 = 3.892, p-value = 0.049) and "communication" versus "skill-based errors" (χ2 = 4.535, p-value = 0.033).

The analysis of the strength of correlation between pairs of human factors indicates that the strength of correlation in all statistically significant relationships is above 0.2. In some of these pairs of human factors such as "national deficiencies" with "MOC", "legislation & regulation" with "PSC", "Communications" versus "inadequate supervision", "legislation & regulation" with "organizational processes", "PSC" versus "failure to solve known problems" and "PRF" with "inadequate planning" the correlation strength exceeds 0.3 (Φc = 0.360, Φc = 0.414, Φc = 0.322, Φc = 0.310, Φc = 0.301, Φc = 0.323 respectively).

The results of the odds ratio between adjacent levels in HFACS-MEs using logistic regression are presented in Table 4. The results showed that defects in rules and regulations at level 5 can increase the probability of creating a weak PSC to 6.94 times and the probability of poor processes in the hospitals to 2.53 times (OR, 6.937; 95% CI, 19.3–2.49 and OR, 2.531; 95% CI, 1.54–8.21 respectively). Additionally, the presence of national deficiencies can elevate the probability of not paying attention to MOC in hospitals to 7.33 times (OR, 7.328; 95% CI, 2.09–25.61).

**Table 4:** The pairs kept by the odds ratio values between different HFACS-MEs levels.

#	Pairwise human factors	OR	β	P_Value	95% CI	
					Lower	Upper
**Relationship between level 5 and Level 4 (5×4)**						
1	National Deficiencies × Management of change	7.328	1.992	0.002	2.096	25.618
2	Legislation & regulation × Patient Safety Culture	6.937	1.937	0.000	2.492	19.307
3	Legislation & regulation × Poor Organisational process	2.531	1.090	0.041	1.542	8.214
**Relationship between level 4 and Level 3 (4×3)**						
4	Management of change × Inadequate planning	4.522	1.509	0.012	1.389	14.721
5	Resource management × Supervisory violation	2.895	1.163	0.048	1.090	8.785
6	Poor Organisational process × Inadequate planning	2.494	1.109	0.049	1.031	6.302
7	Patient Safety Culture × Failure to address a known problem	2.459	1.102	0.045	1.475	7.231
**Relationship between level 3 and Level 2 (3×2)**						
8	Inadequate supervision × Communication	4.750	1.558	0.003	1.673	13.484
9	Inadequate planning × Patient related factors	4.444	1.492	0.003	1.655	11.939
**Relationship between level 2 and Level 1 (2×1)**						
10	Patient related factors × Decision Errors	2.917	1.070	0.030	1.109	7.668
11	Patient related factors × Situational violations	2.985	1.059	0.048	1.080	9.423
12	Person-machine interface × Skill-based Errors	4.286	1.455	0.012	1.371	13.396
13	Physical Environmental × Perpetual Errors	7.194	1.967	0.011	1.480	24.293
14	Chronic performance limitation × Situational violations	4.182	1.431	0.048	1.015	17.234
15	Adverse physiological state × Perpetual Errors	7.200	1.974	0.012	1.550	33.439
16	Adverse mental state × Perpetual Errors	3.283	1.189	0.047	1.1020	11.646
17	Communication × Skill-based Errors	2.944	1.080	.037	1.066	8.135

At casual level 4, ignoring MOC can increase the likelihood of inadequate planning to 4.52 times (OR, 4.522; 95% CI, 1.38–14.72). Defects in resource management can increase the supervisory violations 2.90 times (OR, 2.895; 95% CI, 1.09–8.78). Similarly, weak organizational processes within hospitals can lead to a 2.49 times increase in inadequate planning (OR, 2.494; 95% CI, 6.30–1.03). Additionally, a weak PSC within the organization can increase the probability of failure to address a known problem 2.46 times (OR, 2.459; 95% CI, 1.47–7.21).

At casual level 3, inadequate supervision in hospitals can affect people’s communication and lead to 4.75 times increase in the probability of creating communication defects (OR, 4.750; 95% CI, 1.67–13.48). Additionally, insufficient planning at this level can elevate the probability of PRF-induced errors 4.44 times (OR, 4.444; 95% CI, 1.65–11.93).

The results of the relationship between casual levels 2 and 1 showed that the defects in PRF is one of the factors that can adversely affect decision errors and situational violations and increase the probability of their occurrence 2.92 and 2.99 times, respectively. (OR, 2.917; 95% CI, 1.10–7.66 and OR, 2.985; 95% CI, 1.08–9.42 respectively). Also, creating defects in the person-machine interface can increase the probability of skill-based errors 4.29 times (OR, 4.286; 95% CI, 1.37–13.39). Defects in the physical environment can also increase the perceptual errors 7.2 times (OR, 7.194; 95% CI, 1.48–24.29). The existence of chronic limitations in performance also increases situational violations 4.18 times (OR, 4.182; 95% CI, 1.03–17.23). The presence of harmful physiological states and adverse mental states in health care providers can also increase the probability of creating perceptual errors 7.2 and 3.28 times (OR, 7.200; 95% CI, 1.55–33.43 and OR, 3.283; 95% CI, 1.10–11.64 respectively). Finally, failure in communication between health care providers can increase skill-based errors 2.94 times (OR, 2.944; 95% CI, 1.06–8.13).

**Fig 4:**
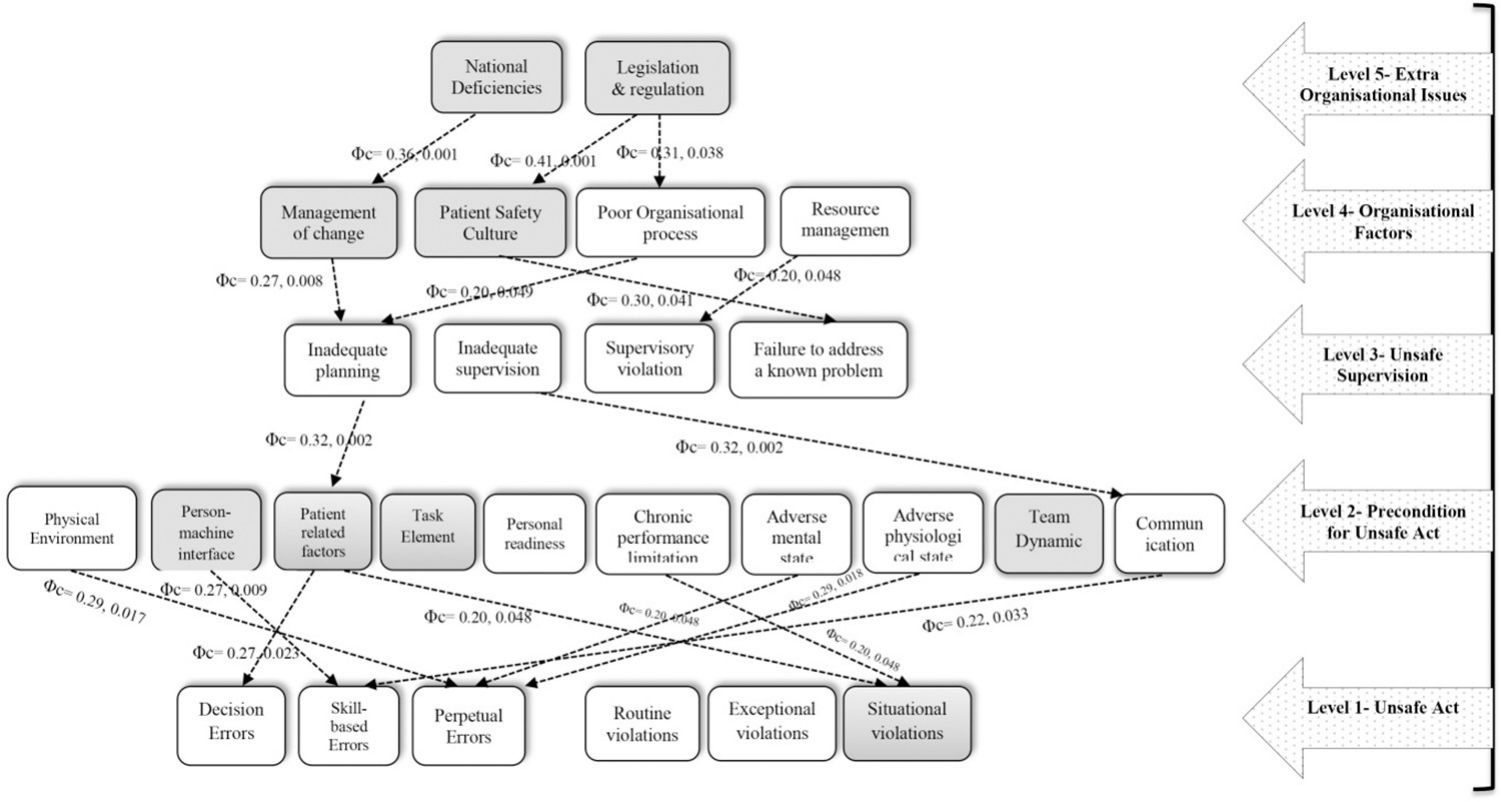
Causality Paths among categories in the HFACS-MEs framework (Φc = Phi and Cramer’s V test, significant associations using χ2).

The screened HOFs among the casual categories of the developed HFACS-MEs model along with the causalities paths are shown in Fig 4. As can be seen, level 5 causal categories including "national deficiencies" and "legislation & regulation" have statistically significant relationships with causal factors of lower levels. Additionally, the causal category of MOC, as one of the new factors added to the HFACS-MEs framework, is affected by the higher causal category (national deficiencies) and has an effect on a lower causal class (inadequate planning). Furthermore, the newly introduced causal category of PRF is significantly influenced by the higher level (through inadequate planning) and has an effect on the lower-level causal categories (demonstrating a statistically significant impact on decision errors and situational violations). Similarly, as one of the newly incorporated factors into the HFACS-MEs framework, the causal category of "situational violations" is influenced by the causal categories of "chronic performance limitation" and PRF.

## Discussion

This study introduces, for the first time, an improved framework for analysis of human factors and their role in MEs, so-called HFACS-MEs, which has been systematically developed and validated using a comprehensive approach. The framework emerged through a Delphi study, leveraged by insights from HFACS developments in various industries and extensive experience and expert knowledge. HFACS-MEs model comprises 5 causal levels and 25 causal categories. Specifically, the model introduces one new causal level and six additional causal categories in comparison to the original HFACS model. Key modifications included the incorporation of the fifth causal level termed "extra-organizational issues," alongside the inclusion of the causal categories "legislation & regulation" and "national deficiencies". Moreover, MOC was introduced, and PSC replaced "organizational culture". Notably, PRF and "task elements" were added, and "person-machine interface" was substituted with "technological environment". Furthermore, transformations in the "personal factors" category resulted in the establishment of "communication" and "team dynamics" as separate categories. Finally, the addition of "situational violations" to the "unsafe act" causal level represented a pivotal change in HFACS-MEs compared to the original HFACS. The framework’s efficacy in revealing causal pathways of MEs was successfully demonstrated through the analysis of 180 MAEs. The outcomes unequivocally indicated that the newly incorporated causal levels and categories played a significant role in causing MEs, exhibiting statistical associations with the levels and causal categories subordinate to them, despite their absence in the original HFACS.

In the course of this study, an additional fifth causal level was integrated into the HFACS framework subsequent to a comprehensive review of the literature. In the initial Delphi round, this level was redefined as "Extra-organizational issues", encompassing two fundamental causal categories: "legislation & regulation" and "national deficiencies". An analysis of the interplay between this new level and the "organizational factors" level unequivocally underscored its significance. Notably, from a pool of eight potential relationships, three pairs of associations emerged as statistically significant between the categories situated at these adjacent levels. In the category of "legislation & regulation", the review generally covers aspects such as the "status of national regulations protecting HCS personnel in case of MEs", and "the status of national and international standards aimed at preventing MEs and enhancing patient safety". Under the category of "national deficiencies", nanocodes such as "the medical and technical level of countries", "national shortage of some specialties" and "national limitations in access to some equipment and facilities" are mentioned. Clearly, these aspects transcend the purview of individual hospital organizations and lie beyond their direct control. The structure and framework governing patient safety, along with overarching policies for ME prevention, operates at levels beyond the scope of senior hospital management, governed by national governments and international bodies like the World Health Organization (WHO). Lapses in such structures and insufficient emphasis on improving patient safety can significantly impact hospitals and healthcare systems globally [27]. Particularly, the shortcomings in international patient safety standards are noticeable. The issue of global patient safety emerged relatively recently, with the first global initiative for patient safety launched in 1999 following the Institute of Medicine’s report titled "To Err Is Human". Despite notable progress, patient harm continues to be a persistent challenge across healthcare systems worldwide [6]. Nonetheless, the WHO introduced a practical guide in 2021, the ’Global Patient Safety Action Plan 2021–2030’, serving as a promising step towards enhancing patient safety and averting MEs. Recognizing the deficiencies in "legislation & regulation" at the highest echelons of government and the absence of overarching laws at the national level safeguarding healthcare providers and the HCS affected by MEs, it becomes imperative to elevate this aspect to the original HFACS framework. The interplay of the causal categories within this tier with "organizational factors" further underscores this imperative. The significance of these factors has been highlighted in various other industries, where the utilization of such tiers within the HFACS framework is well-established [19,20,27].

At the fourth causal level, the initial round of the Delphi study saw the elimination of "organizational climate" by the experts, favouring the utilization of "organizational culture" as a more fitting alternative. However, in the subsequent round, an overlap was identified between "organizational culture" and PSC, prompting the decision to retain PSC exclusively and discard "organizational culture". "Organizational culture" constitutes a composite of values, beliefs, behaviours, customs, and attitudes that underpin the conduct of individuals within organizations. On the other hand, "organizational climate" denotes the collective perception and sentiment concerning the culture within an organization. Essentially, the culture embodies the authentic ethos of the organization, correlating with its macro-level perspectives, while the organizational climate reflects the perceptions of individuals, relating to the micro image of the organization. Given that the fundamental objective within the HFACS framework involves discerning the genuine essence of the organization in relation to its safety culture, the adoption of "organizational culture" in lieu of "organizational climate" appears more pertinent [37,38]. Peerally et al. [23], also used safety culture instead of safety climate in their study on serious hospital incidents. In other studies, these two have been used interchangeably [15,21,39]. As mentioned, within the Delphi study, the causal category ’organizational culture’ was replaced by PSC. "Safety culture" and "organizational culture" represent interconnected concepts often used interchangeably. While ’safety culture’ encompasses facets related to communication and collaborative dynamics among providers, "organizational culture" pertains to the presence of hierarchies and authority gradients. Nonetheless, in the context of examining HOFs, both underscore cultural aspects linked to enhancing patient safety and patient care services [40]. PSC has been proposed as a concept that prioritizes the organizational dimensions of patient safety [41]. Owing to its emphasis on organizational aspects, PSC is found to be more operationally feasible in studies concerning patient safety and the prevention of MEs [42,43]. Consequently, the decision to employ PSC over "organizational culture" in this study was driven by expert opinions and supported by scientific literature.

The introduction of MOC as a contributing factor to MEs was another novel addition in this study. In the context of HCS, MOC encompasses the examination of hardware and software modifications, alterations in processes, changes in operational protocols, and organizational adjustments [44]. Recent research has delved into the significance of leadership and MOC as a pivotal aspect of the role of nurse managers [45–47]. The capability to adapt and embrace changes is vital for delivering contemporary healthcare services, catering to the population’s evolving needs, increasing life expectancies, and managing complex health conditions. Consequently, HCS should recognize MOC as a fundamental competency for healthcare leaders and managers [44]. Inadequately communicated changes and mismanaged transitions can set the stage for accidents and mishaps [19,48]. Most significantly, the costs of mishandling changes may extend beyond the failure of patient safety initiatives, encompassing severe staff hesitancy in response to calls for improved patient safety [48]. Consequently, the neglect of MOC at the organizational level stands out as a critical shortcoming in the original HFACS, necessitating its inclusion in incident analysis, particularly in the context of MEs. This observation is consistent with other studies that have underscored the oversight of MOC in the original HFACS framework as a notable deficiency [19,20]. The cause-effect analysis in the present study also confirms the changes presented at the causal level of "organizational factors" of HFACS-MEs. MOC was notably influenced by the higher-level causal category (national deficiencies) and exerted an impact on the lower-level causal category (inadequate planning). Weaknesses in PSC exhibited a robust association with "failure to address a known problem". The significance of these causal categories is underscored by the observation that the disregard for MOC increased the likelihood of "inadequate planning" by 4.5 times, while a deficient PSC raised the likelihood of "failure to address a known problem" by 2.5 times. These results are in line with other causality analysis studies in the framework of HFACS. George et al. [36], stated that "inadequate safety culture" at chemical industries can increase "failure to address a known problem" to 2.3 times. Chen [20], observed in the construction industry a statistically significant relationship between not paying attention to MOC and "inadequate planning". Theophilus et al. [19], found that there is a statistically significant relationship between lack of attention to MOC and "inadequate supervision" in the oil and gas industries. Furthermore, the current study revealed significant relationships between deficiencies in "resource management" and "supervisory violations", and between shortcomings in "organizational processes" and "inadequate planning", which is in line with the study of Li et al. [49] in the military aviation industry and the study of Jiang et al. [50] in the storage of hazardous chemicals.

One of the significant modifications in HFACS-MEs was the incorporation of PRF into the "environmental factors" causal category. The term "technological environment" was revised to "person-machine interface", while the causal category of "personal factors" was redefined as "team & coordination", comprising two distinct components: "communication" and "team dynamics". Furthermore, the addition of "task elements" to the causal class of "environmental factors" represented another change introduced during the Delphi study. PRF encompasses aspects associated with both the patient and their companions, stemming from anatomical issues, mental health concerns, and conditions like depression or substance abuse. Factors such as the simultaneous presence of two diseases (such as obesity and smoking, which may impact treatment options), non-adherence to the care plan, reluctance to cooperate with healthcare providers, fear of medical services or the hospital environment, communication barriers (including language or accent differences), and lack of cooperation from the patient’s companions were all considered within the scope of PRF. All of these factors contribute to MEs but are often beyond the control of HCS. Neglecting to address PRF can significantly impede hospitals’ ability to manage the resulting repercussions. The significance of this causal class becomes more apparent when it is influenced by the higher causal category ("inadequate planning") and subsequently impacts lower causal categories (statistically significant effects on "decision errors" and "situational violations"). Flaws in PRF can have an adverse impact on "decision-making errors" and "situational violations", thereby increasing the likelihood of their occurrence 2.91 and 2.98 times, respectively. Consequently, focusing on this causal category and incorporating which in the study of the causality of MEs is highly beneficial, highlighting a critical deficiency in previous studies that have employed the original HFACS to determine HOFs leading to MAEs [15,51–53]. Peerally et al. [23], in a study on serious hospital events used the PRF in their HFACS framework and found this factor to be the cause in 20% of events. In another study, Thiels et al. [21], identified "patient-related factors" in 12% of identified nanocodes at the causal level of preconditions for unsafe practices. Woodier et al. [24], also considered PRF as a factor in the HFACS framework in a study in acute hospitals. The consistency of these findings with the present study underscores the significance of incorporating this causal category into the new HFACS-MEs framework.

The "technological environment" stands as one of the crucial causal categories that significantly contribute to accidents. Its role as a key factor in adverse medical events has been extensively documented [28,51,54]. Within the original HFACS, the "technological environment" encompasses various aspects, including equipment and controls design, display/interface features, checklist layout, task elements, and automation. However, in this study, this factor was replaced with "person-machine interface" based on expert opinions and prior scientific literature [22]. Experts suggested that the use of technological environment" may inadvertently divert attention away from factors such as task elements and physical ergonomics. Consequently, the "technological environment" was split into two causal categories, namely "task elements" and "person-machine interface". This division at the causal level of "preconditions for unsafe acts" is expected to streamline the diagnosis of MEs in the causality analysis process, although further studies are warranted to validate this claim. The findings from the causality analysis demonstrated a statistically significant correlation between "person-machine interfaces" and "skill-based errors", with a defect in this area resulting in a 4.28-fold increase in the likelihood of "skill-based errors". These results were consistent with the results of Jiang et al. [50], on hazardous chemical reservoirs, where the technological defects were shown to be capable of increasing "skill-based errors" 9 times. A poor technological environment may make it hard for workers to familiarize themselves with important equipment and operational details, leading to "skill-based errors".

In another modification within HFACS-MEs, the expert panel members renamed the causal category "individual factors" to "team & coordination" and endorsed two subcategories labelled "communication" and "team dynamics" under this category. "Crew resource management" is an aviation-specific term typically used to describe issues such as inadequate information exchange and insufficient teamwork between aircraft and air traffic control during mission execution. Consequently, in the original HFACS, "crew resource management" essentially pertains to challenges related to team communication and coordination [22]. In HCS, inadequate information exchange among managers, supervisors, or healthcare providers, along with ineffective collaboration between teams, can also give rise to unsafe acts. Thus, employing designations like "communication" and "team dynamics" aptly captures the intricacies of healthcare services. These findings resonate with the research outcomes of Peerally et al. [23] in hospital care, Woodier et al. [24] in acute hospitals and Jiang et al. [50] in the storage of hazardous chemicals. Furthermore, the examination of causal relationships at levels two and one distinctly highlights the correlation between defects in "communication" and "team dynamics" with "skill-based errors". Deficiencies in "communications" among healthcare providers can elevate "skill-based errors" by 2.94 times. Previous studies have consistently underscored deficiencies in "communication" and "teamwork" among managers, supervisors, and healthcare providers as one of the principal contributors to MEs. Neuhaus et al. [28], stated communication and coordination deficiencies as one of the most important causes in critical incident reports. Cohen et al. [1] in various healthcare departments, Hsieh et al. [55] in emergency departments and Diller et al. [15] in HCS had found defects in "communication" and "teamwork" as one of the most important causes of MEs.

Introducing the causal class of "situational violations" to the causal level of "unsafe acts" marked the final alteration made in HFACS-MEs. Drawing from the original HFACS, one of the pivotal causal categories in the "unsafe acts" causal level is "violations", encompassing "routine violations" and "exceptional violations". In addition to these categories, several studies have suggested the inclusion of another type of violation known as "situational violations" within this group [56–58]. "Situational violations" arise when workplace conditions compel or entice employees to contravene specific regulations [56]. Within this study, "situational violations" comprised instances of time pressure, inadequate staffing (mismatch between employee numbers and workload), limited access to necessary equipment, and equipment saturation at specific times due to scarcity. Incorporating this causal category enables a more comprehensive classification of violations. The correlation of this causal category with ’chronic performance limitation’ and PRF further validates its inclusion in HFACS-MEs. It appears that healthcare providers may engage in such violations under the influence of PRF, including patient and companion conditions, and due to the presence of "chronic performance limitation". The utilization of this classification in HFACS frameworks tailored for other industries has been underemphasized and rarely applied. In a study, Yang et al. [29] presented a new HFACS framework for process industries, gas, oil and power plants, in line with the results of the present study, in which they used five classifications to determine violations, one of which being situational violations.

Revealing the causal pathways in the genesis of MAEs constituted one of the most critical outcomes of the present study, which not only authenticated HFACS-MEs but also furnished us with valuable insights pertaining to MAEs. This study marked the inaugural investigation into the role of "extra-organizational issues" in MAE genesis, with the causal pathways evidently delineating the impact of causal categories at this level on the lower strata. Notably, the influence of "national deficiencies" on MOC deserves mention. It appears that "national deficiencies" can culminate in the neglect of MOC within organizations, consequently leading to "inadequate planning". Inadequate planning also emerged as a primary instigator of oversight of PRF, contributing to heightened occurrence of "decision errors" and "situational violations". The pivotal role of PRF in numerous MAEs underscores its paramount significance. Moreover, the trajectory stemming from ’legislation & regulation’ was equally intriguing. This path underscored the loopholes in the external "legislation & regulation" which lie beyond the purview of hospitals but could adversely impact the organization’s PSC. Furthermore, flaws in "organizational processes" also stemmed from these "legislation & regulation" inadequacies, fostering negative implications for the organization’s supervisory capacities, including "inadequate supervision" and "inadequate planning". The presence of these latent gaps could engender glaring errors such as "skill-based errors" by impinging upon the "communication" and "teamwork" among managers, supervisors, and healthcare providers. As is widely recognized, the identification of causal pathways encompassing all HOFs stands as one of the salient merits of the HFACS method, which here has been enhanced due to targeted modifications. This phenomenon has been well established and corroborated by other researchers across various studies conducted in diverse industries [19,20,29,49,50].

In the present study, Delphi method was used to determine the most important and relevant factors in creating MAEs. This Delphi study was completed after two rounds and based on its results, the causal levels and categories of the HSACS-MEs framework were developed. The results of the causal analysis also indicated that by using the changes made in the original HFACS, some limitations in the analysis and finding the causes of MAEs can be resolved. The application of this framework has demonstrated the role of the newly added causal categories in contributing to the occurrence of MAEs, showcasing their interrelation with lower causal levels and categories. These results indicated the appropriate output of the Delphi method in determining HOFs related to MAEs. The use of Delphi method has recently been widely used in health sciences and determining factors affecting MEs. In a study, Mousavi et al. [59], used the Delphi technique to determine and prioritize factors affecting the occurrence of needle stick injuries in health workers. In another study, Ghasemi and Taheri [60] used the Delphi method to determine the factors influencing the creation of MEs and stated that this method can well bring the opinion of experts to a consensus on specialized issues. Determining the factors affecting MEs in other studies have also been well determined using Delphi and is in line with the results of the present study [61–64].

This study has limitations that should be considered and addressed in future studies. Firstly, the Delphi method was used to determine the HOFs of the new HFACS-MEs framework. Delphi studies often rely on expert opinions to generate findings. As a result, the quality of evidence may be affected if experts are weak. However, given that the HOFs were derived from a comprehensive literature review, particularly studies on HFACS development in various industries, the findings are likely reliable. Secondly, we retrospectively performed causality with the HFACS-MEs framework. Some studies believe that the retrospective use of HFACS should be accompanied by some limitations [15]. However, in the present study, the defects and ambiguities in the RCA reports were resolved in two ways: First, getting help from the RCA committee members of the hospitals, and second, by attending and visiting the hospitals.

Given that, HFACS is focused on the examination of human factors and relies on human elements, it is susceptible to evolving with time. Factors such as technological advancements, treatment methodologies, work protocols, tools, and work environments have undergone considerable transformations from the past to the present, warranting the contemporary emphasis on MOC compared to earlier times. Thus, in this study, it was modified to the so-called HFACS-MEs. Therefore, HFACS-MEs must be continuously improved to be applicable to emerging and updating MEs. Also, the proposed HFACS-MEs boasts more causal levels and categories than the original HFACS, potentially intensifying the method’s complexity. Future investigations are suggested to delve into this matter during practical applications. Finally, this study employed the χ2, Φc, and OR to probe causal pathways. These techniques only establish connections between contiguous levels and do not ascertain the simultaneous interactions among all the factors across all the levels and their impact on the overt levels (unsafe acts level) in a singular model. The use of dependence-based methods such as Bayesian network is suggested to predict the probability of occurrence of unsafe human factors. Furthermore, due to the heightened workload of healthcare personnel in hospitals, there may be insufficient time to utilize HFACS-MEs for identifying the causes of MEs and MAEs. Therefore, in future studies, it is recommended to simplify this framework and reduce its complexity to save personnel time and promote wider utilization.

## Conclusion

In this study, a new framework for the analysis of human factors in medical errors, so-called HFACS-MEs, was presented and validated with a systematic and combined approach. The findings suggest that the original HFACS framework is inadequate for the analysis of MAEs. The incorporation of a new causal level "extra-organizational factors" (comprising the causal categories of "legislation & regulation" and "national deficiencies") alongside the inclusion of causal categories such as "management of change", "patient safety culture", "patient related factors", "task elements" and "situational violations" has proven instrumental in resolving certain limitations in analysis and diagnosis of MEs. The application of this framework has demonstrated the role of the newly added causal categories in contributing to the occurrence of MAEs, showcasing their interrelation with lower causal levels and categories. The framework developed in this study serves as a valuable tool in identifying the causes and causal pathways of MAEs, facilitating a comprehensive analysis of the human factors that significantly impact patient safety within healthcare settings.
